# The impact and complete genome characterisation of viruses involved in outbreaks of gastroenteritis in a farrow-to-finish holding

**DOI:** 10.1038/s41598-023-45994-4

**Published:** 2023-10-31

**Authors:** Dragan Brnić, Dunja Vlahović, Andrea Gudan Kurilj, Nadica Maltar-Strmečki, Ivana Lojkić, Valentina Kunić, Lorena Jemeršić, Ivica Bačani, Gordan Kompes, Relja Beck, Tina Mikuletič, Andrej Steyer

**Affiliations:** 1 Croatian Veterinary Institute, Savska cesta 143, 10000 Zagreb, Croatia; 2 Department of Veterinary Pathology, Faculty of Veterinary Medicine, University of Zagreb, Heinzelova 55, 10000 Zagreb, Croatia; 3 Laboratory for Electron Spin Spectroscopy, Division of Physical Chemistry, Ruđer Bošković Institute, Bijenička cesta 54, 10000 Zagreb, Croatia; 4 Animal Feed Factory, Dr Ivana Novaka 11, 40000 Čakovec, Croatia; 5 Institute of Microbiology and Immunology, Faculty of Medicine, University of Ljubljana, Zaloška 4, 1000 Ljubljana, Slovenia; 6 Division of Public Health Microbiology, National Laboratory of Health, Environment and Food, Grablovičeva 44, 1000 Ljubljana, Slovenia

**Keywords:** Clinical microbiology, Virology

## Abstract

Viral enteric pathogens continuously burden intensive pig farming, causing gastrointestinal diseases of epidemic and endemic nature. The present study investigated two diarrhoea outbreaks on a large farrow-to-finish holding and subsequent circulation of outbreak-related enteric viruses. These viruses were characterised by whole genome sequencing, and statistical evaluation of the impact on specific production metrics was performed. The results provided evidence that the *Porcine epidemic diarrhoea virus*–swine enteric coronavirus (PEDV–SeCoV) S gene recombinant strain was responsible for the first outbreak, whilst *Rotavirus A* (RVA) in a mixed infection with *Rotavirus B* (RVB) and porcine kobuvirus (PKV) probably caused the second diarrhoea outbreak. Whole genome characterisation revealed a porcine origin of all viruses involved and significant heterogeneity of RVB strain, proposing four novel genotypes and changes in RVB VP1 genotype classification. The statistical evaluation confirmed only a minor disturbance in the number of weaned pigs per sow, with statistical forecasting showing positive trends. A follow-up study corroborated the endemicity of RVA and PKV, in contrast to PEDV–SeCoV. Punctual, comprehensive and timely investigation of diarrhoea outbreaks is a prerequisite for applying adequate pig health and biosecurity management. Calculating such outbreaks' impact on production metrics can potentially shape future decisions on management improvements.

## Introduction

Viral gastroenteritis is one of the most impactful diseases in intensive pig production. Different causative agents are continuously emerging/reemerging in the pig population worldwide^1^. Coronaviruses and rotaviruses often stand behind the aetiology of viral diarrhoea in pigs, especially in younger age categories^2^.

Among pigs' most significant enteropathogenic coronaviruses are transmissible porcine gastroenteritis virus (TGEV) and *Porcine epidemic diarrhoea virus* (PEDV). They are positive single-stranded RNA viruses (~ 28 kb in size) of the genus *Alphacoronavirus* within the family *Coronaviridae*^2,3^. While TGEV has become sporadic, primarily due to the extensive prevalence of porcine respiratory coronavirus (PRCV; deletion variant of TGEV), novel PEDV variants (non-S-INDEL and S-INDEL) have emerged worldwide since 2010^4^. Europe was primarily affected by the PEDV S-INDEL strain in 2014 and 2015^5^, also detected in Croatia in 2016^6^. Chimeric strains called swine enteric coronaviruses (SeCoV), possessing the TGEV backbone genome and PEDV S gene, have circulated in Europe since 1993^7–11^. More recently, SeCoVs were involved in recombination events with PEDV strains within the S gene. These PEDV-SeCoV recombinants have recently emerged in Europe^12–14^. All coronaviruses mentioned above were connected to high morbidity with evident clinical symptoms of diarrhoea and vomiting accompanied by low-to-moderate (PEDV S-INDEL, SeCoV, PEDV-SeCoV recombinants) or high mortality rates (TGEV, PEDV non-S-INDEL)^4,10^, mainly in suckling piglets. However, more information is needed about the impact of PEDV-SeCoV recombinants on production results in large holdings.

Rotaviruses (RV) are highly environmentally resistant and ubiquitous, which makes pig health management demanding^15^. These viruses (~ 18.5 kb in size) possess a double-stranded segmented (N = 11) RNA genome and are members of the *Reoviride* family, genus *Rotavirus*^3,16^. Out of nine officially recognised species (*Rotavirus A–D* and *Rotavirus F–J*)^16^, four are known pathogens in pigs, *Rotavirus A* (RVA*), Rotavirus B* (RVB), *Rotavirus C* (RVC) and *Rotavirus H* (RVH)^17^. In addition to the standard binomial nomenclature of RV genotypes (VP7 and VP4 segments denominating G and P genotypes, respectively), the whole-genome-based genotype classification system has been established, more precisely Gx-P[x]-Ix-Rx-Cx-Mx-Ax-Nx-Tx-Ex-Hx designate VP7-VP4-VP6-VP1-VP2-VP3-NSP1-NSP2-NSP3-NSP4-NSP5 genomic segments^18^. Rotaviruses in domestic pigs are highly genetically diverse, especially RVA, the most investigated RV species^19^. That was also evidenced in our latest study performed during three consecutive RVA seasons (2018–2021), where we detected eight and seven different G and P genotypes, respectively, in 23 different genotype combinations^20^. In general, rotaviruses in pigs are underinvestigated, especially considering species other than RVA with a lack of whole genome-based studies^17,21^. The clinical outcome of RV infections (indistinguishable from coronavirus infections) ranges from subclinical to severe, resulting in low-to-moderate mortality, mostly in suckling piglets^15^.

Apart from coronaviruses and rotaviruses, many other viruses are involved in the aetiology of neonatal diarrhoea in pigs^22^. For instance, porcine kobuvirus (PKV), classified as *Aichivirus C* species in the *Kobuvirus* genus within the *Picornaviridae* family^23^, is considered a potential causative agent of diarrhoea in pigs^24^, even though its importance as a sole pathogenic agent is debatable^25^. The genome of PKV is a single-stranded positive-sense RNA, only ~ 8.2 kb in size^3^. It circulates in high prevalence in diarrhoeic and healthy pigs^25^.

The present study aimed to investigate the aetiology and impact of two outbreaks of diarrhoea in a large farrow-to-finish holding followed by elevated mortalities in suckling piglets. Subsequent circulation of outbreak-related enteric viruses was further investigated in a follow-up study. The effect on some production results was measured. Furthermore, the complete genome characterisation of the *Porcine epidemic diarrhoea virus*, *Rotavirus A*, *Rotavirus B* and porcine kobuvirus was revealed.

## Materials and methods

### Sampling

During the first half of 2017, the Croatian Veterinary Institute (HVI) was called to assist in resolving two contagious diarrhoea outbreaks (outbreak 1 in January and outbreak 2 in June) causing elevated mortalities in suckling piglets bred on a large farrow-to-finish holding (1000 sows) in Međimurje County (Northern Croatia). The holding did not implement any vaccination scheme to prevent losses from diarrhoea-causing pathogens. In outbreak 1, diarrhoea, vomiting and mortalities were reported in suckling piglets and diarrhoea, vomiting and agalactia in sows. In outbreak 2, diarrhoea and mortalities were reported in suckling piglets. In January 2017, when outbreak 1 was reported, two faecal samples and three piglet carcasses (age 7–10 days) were sent to the HVI and the Department of Veterinary Pathology at the Faculty of Veterinary Medicine, the University of Zagreb (VEF), respectively. In March 2017, following outbreak 1, three rectal swabs of weaned pigs with diarrhoea were collected and sent to the HVI. In June 2017, when outbreak 2 was reported, four piglet carcasses were sent to the VEF for a necropsy.

The affected holding was further monitored for the circulation of viral pathogens confirmed in both outbreaks in a follow-up study from October 2018 until January 2021. Within that period, 67 rectal swabs were sampled from suckling (N = 55) and weaning (N = 12) pigs and sent to HVI. In total, 79 samples were collected from the affected holding, out of which 84.8% (67/79) originated from pigs with diarrhoea.

### Gross and histopathological examination

A routine necropsy examination of seven piglets from two outbreaks (three from outbreak 1 and four from outbreak 2) was performed. Tissue samples (duodenum, jejunum, ileum, colon and mesenteric lymph nodes) collected during necropsy were fixed in 10% neutral buffered formalin, embedded in paraffin, sectioned in five μm thickness, placed on silanised slides and stained with hematoxylin and eosin (HE) for histopathological examination. Intestinal contents were taken from the small intestines of all seven piglets at the necropsy and sent to HVI to perform virological, bacteriological and parasitological tests.

### Laboratory diagnostic investigations

Due to the anamnestic data given by the veterinarian working at the farm, the focus was primarily on the molecular diagnostics of PEDV, TGEV and RVA. A 20% w/v intestinal content or rectal swab suspensions were prepared in Medium 199 (Sigma Aldrich, USA), vortexed and centrifuged for 5 min at 14.000 × *g*. Supernatants were used as starting material for RNA/DNA extraction, which was obtained on KingFisher™ DuoPrime or Flex purification system (Thermo Fisher Scientific, USA) using MagMAX™ CORE Nucleic Acid Purification Kit (Thermo Fisher Scientific, USA) by following the manufacturer's instructions (complex workflow). The exogenous Internal Positive Control (IPC) RNA, Xeno™ RNA Control (Thermo Fisher Scientific, USA), was added to each sample (2 µL) to monitor the appearance of potential PCR inhibitors. The extracted RNA was stored at − 80 °C if not processed immediately.

Detection of PEDV was conducted on two targets, S gene real-time RT-PCR^26^ and N gene Sybr Green real-time RT-PCR (unpublished primers, kindly provided by Dr Akbar Dastjerdi, APHA, UK)^6^. TGEV and RVA real-time RT-PCR protocols were focused on detecting the N gene^27^ and VP2 genomic segment^28^, respectively. All TaqMan probe assays (PEDV S gene, TGEV and RVA) were performed using VetMAX™-Plus One-Step RT-PCR kit (Thermo Fisher Scientific, USA) and following manufacturer's instructions on reaction set-up and cycling. For all these assays, the final concentration of each primer and TaqMan probe was 600 nmol and 200 nmol, respectively. Due to the dsRNA genome, the real-time RT-PCR set-up for the RVA was divided into denaturation and reaction mixture, as described previously^29^. The QuantiTect Sybr Green RT-PCR kit (Qiagen, Germany) was used for PEDV N gene detection (final primer concentration 500 nmol) in line with the manufacturer's instructions on reaction set-up and cycling. All real-time RT-PCR runs were performed on a Rotor-Gene Q or QIAquant 96 5plex (Qiagen, Germany).

Routine parasitological and bacteriological examinations were used to screen the intestinal contents taken at the necropsy of seven piglets for certain parasites (*Isospora suis*, *Cryptosporidium spp* and nematodes) and bacteria (*Clostridium spp.*, *E. coli*, *Salmonella spp.* and *Lawsonia intracellularis*) important from differential diagnostics point of view. Using blood agar and evaluating the presence of haemolysis, the method was able to detect the majority of the enterotoxigenic *E. coli* (ETEC) F4 and F18 strains^30^. Detection of *Lawsonia intracellularis* was performed by PCR using GoTaq® G2 Hot Start Colorless Master Mix (Promega, USA) and previously published primers A and B^31^.

### Next generation sequencing

The NGS was performed on the MiSeq platform (Illumina, USA) on two samples of small intestinal content. First was the sample from outbreak 1 with the lowest Cq value for PEDV S and N gene real-time RT-PCR, and second was the sample from outbreak 2 with the lowest Cq value for RVA VP2 real-time RT-PCR. Library preparation and the MiSeq run were conducted following previously described procedures^32^. The raw data were analysed using Geneious 8.1.8. software (Biomatters Ltd., New Zealand). After initial data quality control and trimming, virome analysis was performed by Kraken 2^33^ and visualised in Krona^34^. Based on virome analysis from the previous step, trimmed reads were mapped to reference genomes of PEDV, RVA, RVB and PKV obtained from the GenBank (PEDV: KU297956, RVA: KX988264-KX988274 and MK936375-MK936425; RVB: KX362400-KX362410 and PKV: KP144318). The mapping was further performed to reference genomes of some other relevant porcine enteric viral pathogens, i.e. astrovirus, *Rotavirus C* and sapovirus (astrovirus: LC201612, RVC: MT874983-MT874993, MG451776, MG4561777, MG451781 and sapovirus: MK962339). Additionally, de novo assembly was utilised on Geneious 8.1.8. (Biomatters Ltd., New Zealand) software with default settings to assemble all RVA and RVB gene segments due to significant genotype diversity. Consequently, the contigs generated by the de novo assembly were compared for similarity against the whole NCBI GenBank virus nucleotide database using BLASTn. Finally, consensus sequences of PEDV, RVA, RVB, and PKV genomes were analysed for their ORFs using Geneious 8.1.8. software (Biomatters, Ltd., New Zealand), and the deduced amino-acid sequences were obtained.

### Follow-up study

All samples taken between 2018 and 2021 (N = 67) and 12 in 2017 during initial outbreaks (intestinal contents from seven carcasses and five faecal/rectal swab samples) were screened for the circulation of the pathogens detected by NGS. We have designed two additional primer sets to detect RVB and PKV based on sequences of corresponding strains determined by NGS in outbreak 2. RVB detection was conducted by primers RVB-VP6-F (5’-TCTGATCGAGACAGTGAATGC-3') and RVB-VP6-R (5’-CTGTGAACTACCTGCTCAATG-3') amplifying 498 bp RT-PCR product of RVB VP6 genomic segment (I9 genotype). PKV detection was performed by primers PKV-3D-F (5’-TGATTCACACTCTGACAATG-3') and PKV-3D-R (5’-CGAGATGTTTCTCAACAATG-3') amplifying 507 bp RT-PCR product of PKV 3D gene. Both assays were performed with the utilisation of SuperScript™ III One-Step RT-PCR System with Platinum™ Taq DNA Polymerase (Thermo Fisher Scientific, USA) with reaction set-up as recommended by the manufacturer and the final primer concentration of 600 nmol. Due to the dsRNA genome of the RVB, the reaction mixture was composed of denaturation and reaction mixture. The RNA sample, RVB-VP6-F primer and PCR-grade water were included in the denaturation mixture, followed by incubation at 95 °C for 5 min. After denaturation, a reaction mixture of 2X Reaction Mix, SuperScript III RT/Platinum Taq Mix and RVB-VP6-R primer was added. Both RT-PCR assays (for RVB and PKV) were run on the same cycling conditions on Biometra TRIO (Analytic Jena, Germany) or ABI 9700 GeneAmp thermal cycler (Applied Biosystems, USA) as follows: 30 min at 50 °C; 2 min at 94 °C; 40 cycles of 30 s at 94 °C, 30 s at 50 °C and 30 s at 68 °C; 5 min at 68 °C.

Samples collected between 2018 and 2021 (N = 67) were screened for PEDV (S and N genes), TGEV (N gene), RVB and PKV. RVA detection (VP2 genomic segment) and VP7/VP4 genotyping were performed on those samples within our previous study (19). Since 12 samples directly related to outbreaks were already screened for PEDV, TGEV and RVA, they were additionally checked for RVB and PKV.

### Sanger sequencing, genotype assignment, phylogenetic and recombination analysis

The Sanger sequencing was used to confirm the specificity of RVB and PKV RT-PCR amplification. RT-PCR products were purified with ExoSAP-IT™ PCR Product Cleanup Reagent (Thermo Fisher Scientific, USA) as described previously^29^. The samples were subjected to direct Sanger sequencing in both directions using Macrogen Europe (Amsterdam, the Netherlands) services.

Genotype assignment of RVA strain was performed by the BLASTn search in combination with the ViPR tool^35^. Cutoff values for each RVA segment were previously defined^18^. The recently updated genotype classification^36^ of RVB strains was combined with the BLASTn to assign a genotype for each RVB segment.

The phylogenetic analysis was done on PEDV, RVA, RVB and PKV strains described in the present study, and a selected number of reference sequences obtained from the GenBank. Multiple sequence alignment was performed by the MUSCLE algorithm, and phylogenetic analysis by the maximum-likelihood (ML) method and models (the lowest BIC score) for each RVA (T92 + G + I for VP7, VP4 and NSP1; T92 + G for VP6 and NSP2-NSP5; TN93 + G + I for VP1; TN93 + G for VP2; GTR + G + I for VP3) and RVB (T92 + G + I for VP7; T92 + G for NSP2; HKY + G + I for VP4; HKY + G for NSP4; TN93 + G + I for VP6 and NSP3; TN93 + G for NSP5; GTR + G + I for VP1-VP3 and NSP1) genomic segment, as well as for the PKV (GTR + G + I). The neighbour-joining (NJ) method combined with the maximum composite likelihood model was used to analyse the PEDV S gene and complete genome. The branching support of the ML and NJ tree was assessed by bootstrap analysis with 1000 repetitions. These analyses were done in MEGA11 software^37^. The phylogenetic trees were visualised and annotated using iTOL^38^. The nucleotide pairwise identity matrix was calculated in BioEdit software, version 7.2.5^39^.

The potential recombination events in the PEDV strain (S gene and complete genome-based) characterised in the present study were analysed using the RDP4 package (version 4.49), which embeds multiple recombination detection methods^40^. Only recombination detected by a minimum of six out of seven methods (RDP, Bootscan, 3Seq, Chimæra, SiScan, MaxChi and Geneconv) was considered valid. The important notice is that "parent" does not necessarily mean the actual progenitor of the recombinant strain but rather a representative of strains whose genome sequences most closely resemble the recombinant^41^.

### Immunohistochemistry

Immunohistochemistry (IHC) was used to detect PEDV, RVA and RVB antigens in tissue samples from all seven piglets from outbreaks 1 and 2. Tissue sections were routinely sectioned (5 μm), placed on silanised slides and heated in a heater at 50 °C overnight. After deparaffinisation and hydration, antigen retrieval was performed with citrate buffer (pH 6.0) in an autoclave for 20 min at 121 °C and 1.1 bar. Endogenous peroxidase activity was quenched with 1% hydrogen peroxide in TBST buffer solution for 30 min. Non-specific binding was blocked with normal goat serum (30 min) before incubation with mouse monoclonal primary antibodies to PEDV (SD6-29, Medgene labs, USA; dilution 1:1000), *Rotavirus A* (3C10, HyTest, Finland; dilution 1:100) and *Rotavirus B* (10B1, Kerafast, USA; dilution 1:100) for 1 h in a humid chamber at room temperature. PKV monoclonal antibodies were unavailable. A peroxidase-conjugated secondary antibody and detection kit was used for visualisation (REAL EnVision Detection System, Peroxidase/DAB+, Rabbit/Mouse kit, Agilent Dako, USA), after which the slides were counterstained with hematoxylin, dehydrated and coverslipped.

### Farm production data

Available time series, i.e., data on piglet mortality, newborns, and weaned pigs per sow, were collected weekly and monthly for three years (2017–2019). Data on the average number of farrowing per sow per year (farrowing index) were also compared. The number of sows for which the calculations were done was 880, 917 and 1017 in 2017, 2018, and 2019, respectively.

### Statistics

Statistical analysis was performed to calculate the significance of the impact of two diarrhoea outbreaks on farm production results. Analyses were performed using Systat V.13.2^42^, and subsequent analysis for prediction was performed in Wolfram Mathematica V.13.0.1.0^43^.

Infectious disease time series are generally characterised by two separate contributions to the underlying pattern. These components, the trend cycle and seasonal factors can usually be identified by decomposition methods. The trend cycle represents long-term changes, while the seasonal factor denotes the periodic fluctuations of constant length^44^.

The seasonal index (SI) expresses the seasonal component of the time series. If the seasonal index is greater than 1 (or 100%), the incidence is higher than the average and vice versa. The SI (%) was calculated by comparing the average number of newborns, mortalities and weaned pigs per sow weekly in 2017 to the average number in the respective week of all months in 2017. Furthermore, the calculation was expanded to compare these data in each month to the average data of the respective month during three years (2017–2019). After the seasonal indices were calculated, the data were deseasonalised by dividing them by the corresponding indices as follows: deseasonalised data = raw data/seasonal index^45^. Then, the Autoregressive Integrated Moving Average (ARIMA) method^46^ was used to estimate the trend based on the 2017–2019 data and to forecast one year ahead (2020). Decisions on statistical relevance were made at the significance level of *p* ≤ 0.05.

### Ethical approval

This study was approved by the Board of Ethics of the Croatian Veterinary Institute (protocol code Z-VI-4–5206/17, approved on 11 December 2017) and complied with ARRIVE guidelines^56^. All applicable international, national, and/or institutional guidelines for the care and use of animals were followed. The pig carcasses were submitted voluntarily by the pig owner and oral consent was obtained prior to sampling.

## Results

### Laboratory diagnostic investigations

Two faecal samples received in January 2017 tested positive for the PEDV S gene (Cq: 9.9 and 11.7) and negative for the TGEV N gene (SeCoV was excluded) and RVA. Small intestinal contents, taken from three piglets at the necropsy, tested PEDV S gene positive (Cq: 11.8, 17.9, 24.8), PEDV N gene positive (Cq: 9.3, 15.8, 23.1; Tm = 83.3 °C) and TGEV N gene negative. RVA VP2 real-time RT-PCR resulted in only one positive sample (Cq: 35.0). Among three rectal swabs collected in March 2017, two tested PEDV S (Cq: 19.4, 29.8) and N (Cq: 17.7, 28.7; Tm = 83.3 °C) gene positive proving the ongoing circulation of PEDV (TGEV was negative). These two PEDV-positive rectal swabs were also RVA-positive (Cq: 25.2 and 33.0).

Small intestinal contents of four piglets received for necropsy in June 2017 tested PEDV (S and N gene) and TGEV negative. However, RVA VP2 real-time RT-PCR tested positive on all four samples (Cq: 11.5, 24.2, 27.8, 30.0).

Successful detection of IPC RNA excluded the presence of PCR inhibitors. The majority of parasite and bacteria species that are important from differential diagnostic point of view were ruled out as potential causative agents.

### Necropsy and histopathology

In all seven piglets, a necropsy showed a small intestine filled with a substantial amount and a large intestine with a smaller amount of watery white-yellowish contents. The intestinal wall, particularly the jejunum and the mesocolon, showed an oedematous appearance. Mesenteric lymph nodes were moderately enlarged.

**Figure 1:**
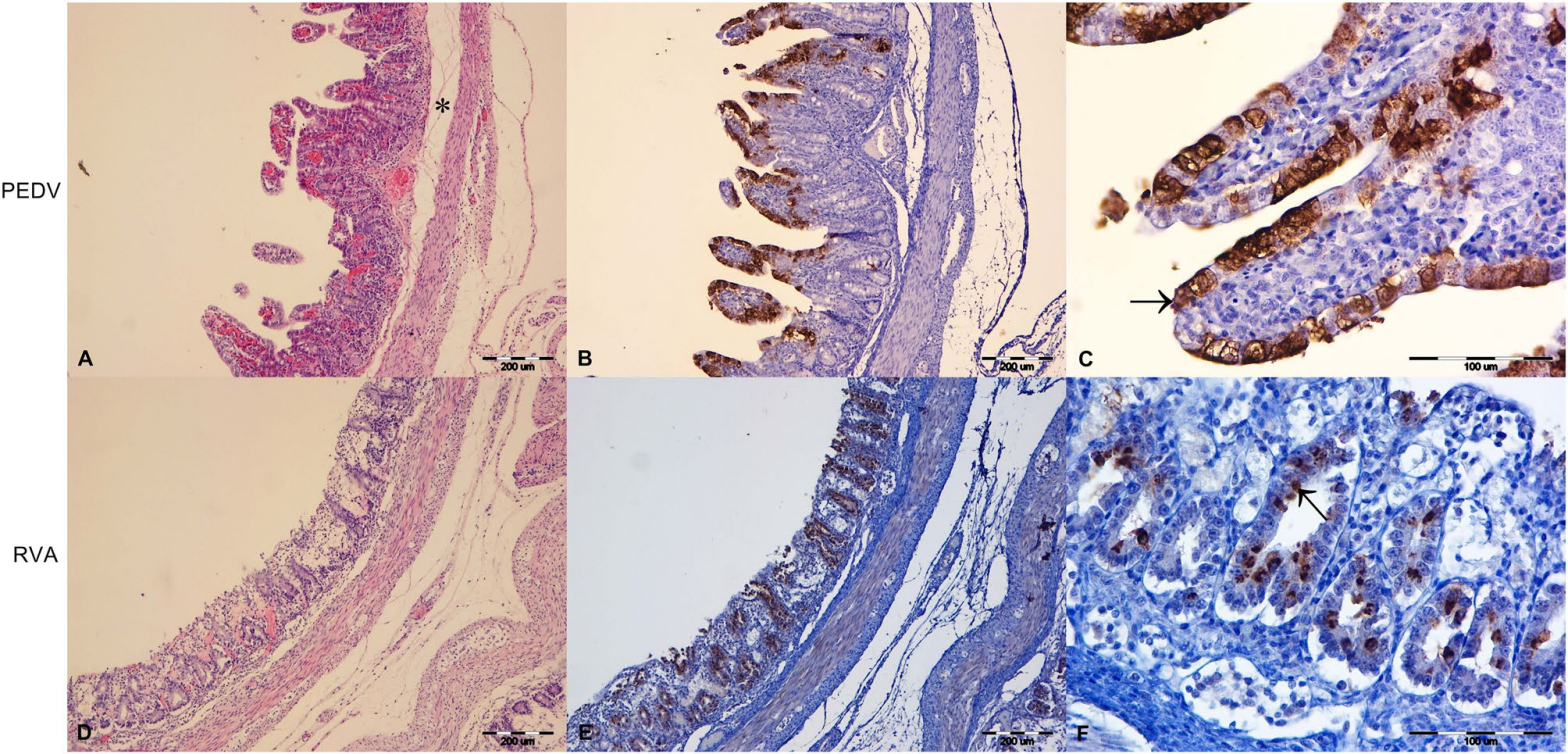
Histopathological and immunohistochemical findings in PEDV (**A**–**C**) and RVA (**D**–**F**) infected piglets. Shortening, blunting and fusion of villi in PEDV-infected pigs were most pronounced in the jejunum, accompanied by marked submucosal oedema (**A**, asterisk) (**A**). In RVA-infected animals, lesions were also present in the colon, characterised by a moderate increase in the number of lymphocytes in the lamina propria, necrosis of individual surface epithelial cells, and oedema of the mesocolon. (HE 10×) (**D**). Positive IHC reaction (brown colour) to PEDV and RVA antigens was detected in the intestinal mucosa (jejunum, **B**, **C** and colon, **E**, **F**) (IHC 10x). Intracytoplasmic brown granular staining was seen in superficial enterocytes in PEDV (**C**, arrow) (jejunum, IHC 40×) and in crypts of Lieberkűhn of RVA infected animals (**F**, arrow) (colon, IHC 40x).

Jejunum was most severely affected with lesions characterised by shortening, blunting and fusion of villi (Fig. 1). Additionally, in PEDV-infected animals, marked submucosal oedema was noticed (Fig. 1). Duodenum and ileum were affected by similar changes but of lesser intensity. Vacuolisation of superficial enterocytes was mainly observed in PEDV-infected piglets. In RVA-infected animals, the lesions were more intense and additionally characterised by stronger enterocyte necrosis and increased lymphocytes in the lamina propria of the small intestine and colon (Fig. 1). Reactive hyperplasia was visible in the mesenteric lymph nodes.

### IHC

Duodenum, jejunum and ileum from all three piglets in outbreak 1 were positive for PEDV antigens. A positive reaction is visible as intracytoplasmic brown granular staining in superficial enterocytes (Fig. 1). Colon and mesenteric lymph nodes were immunohistochemically negative. All samples from four piglets in outbreak 2 were negative for PEDV antigens. All parts of the small intestine (not shown) and the colon from two piglets in outbreak 2 were positive for RVA antigens. Intracytoplasmic brown granular staining was seen in the crypts of Lieberkűhn (Fig. 1). Mesenteric lymph nodes were immunohistochemically negative. All samples from the other two piglets in outbreak 2 and all three in outbreak 1 were negative for RVA antigens. RVB antigens were not immunohistochemically detected.

### NGS, phylogenetic and recombination analysis

The NGS data for the sample from outbreak 1 resulted in 566,026 reads, of which 67,561 were mapped to the PEDV reference genome (KU297956). The mean coverage and Q30 were 388.3× and 99.6%, respectively. The NGS data for the sample from outbreak 2 resulted in 568,064 reads, of which 226,467 mapped to referent RVA strains (KX988264-KX988274, MK936375-MK936425), with the mean coverage 1352× and the Q30 of 96.2%. Furthermore, 49,630 reads were mapped to the referent RVB genome (KX362400-KX362410; mean coverage 250×, Q30 95.9%) and 2037 reads to the referent PKV genome (KP144318; mean coverage 30.7×, Q30 95.3%). The complete CDS were obtained for PEDV, RVA, RVB (except VP2, missing only 2.3%) and PKV. Both de novo and reference mapping assemblies failed to detect the presence of genomes of other viral pathogens in these samples.

**Figure 2:**
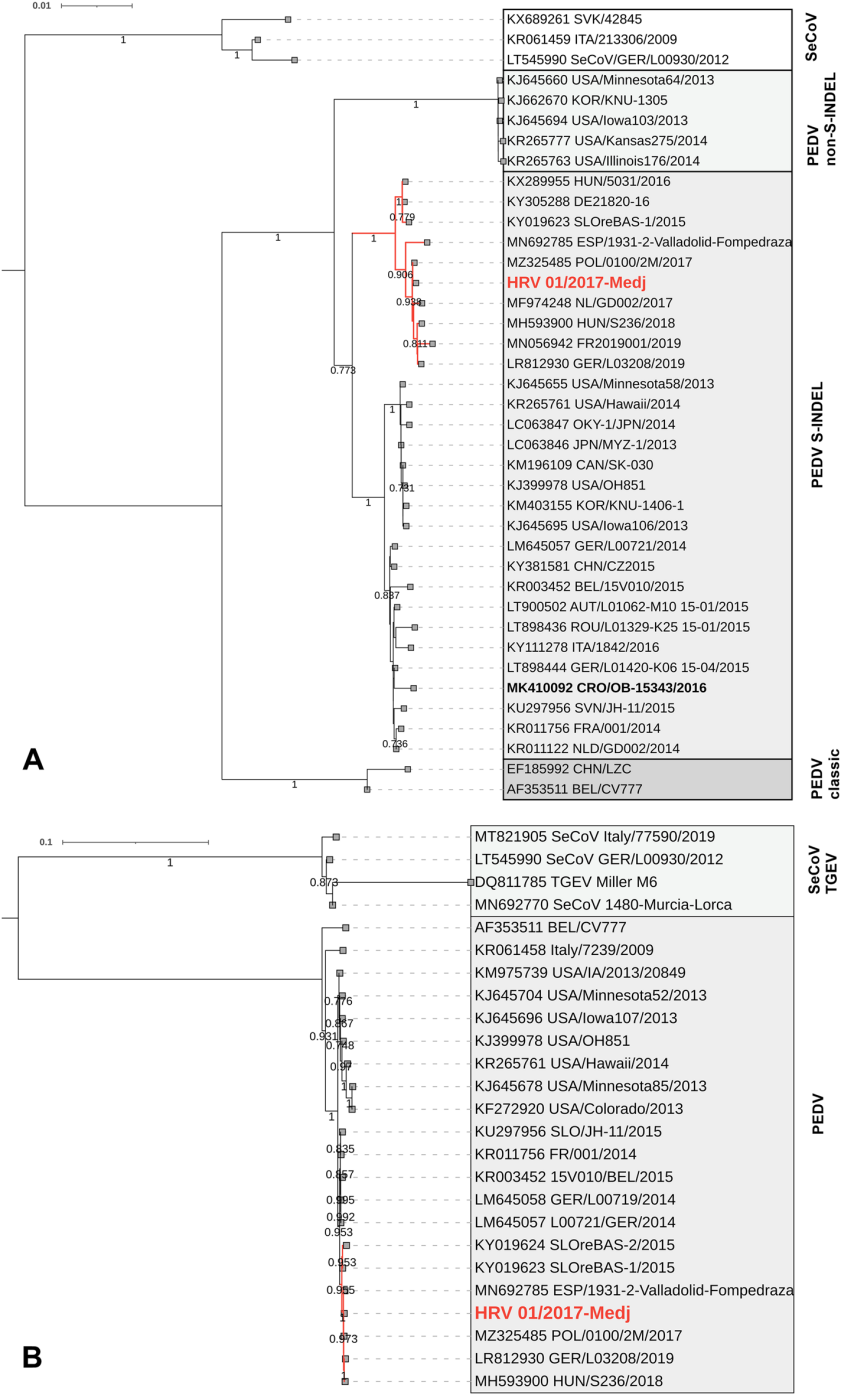
Phylogenetic relationship between PEDV-SeCoV recombinant strain and selected reference strains. The phylogenetic analysis was based on the S gene (**A**) and the complete genome (**B**) nucleotide sequence. The strain described in the present study is bolded and marked in red, while the branch in red designates the cluster of PEDV-SeCoV recombinant strains. The GenBank accession numbers of referent strains are designated within taxa. Both trees were generated by the NJ method and maximum composite likelihood model in MEGA 11 software. The branching stability of each phylogenetic tree was assessed by 1000 bootstrap replicates (values indicated adjacent to the nodes if > 0.7). The scale bar represents the number of substitutions per site.

The PEDV strain responsible for outbreak 1 shared the highest % of nucleotide (nt) and amino acid (aa) identity with the PEDV-SeCoV recombinants reported to circulate in Europe at that time (Fig. 2). The highest identity was with the Polish strain (MZ325485) on the S gene and whole genome level (99.9% on nt and aa levels). The backbone genome originated from the S-INDEL PEDV strain (Fig. 2), with recombination detected only in the S gene by all seven RDP4 methods. The breakpoint positions at 225 (99% CI 208–231) and 636 (99% CI 575–653) of the S gene nucleotide sequence (410 nt long stretch from SeCoV) were identified. The strains KU297956 (SVN/JH-11/2015) and KX689261 (SVK/42845) were defined as major and minor parents, respectively. The current PEDV strain (HRV 01/2017-Medj) and the Croatian PEDV strain reported in 2016 (CRO/OB-15343/2016)^6^ clustered separately (Fig. 2) and shared the S gene nt and aa sequence identity of 98.5% and 96%, respectively.

**Figure 3:**
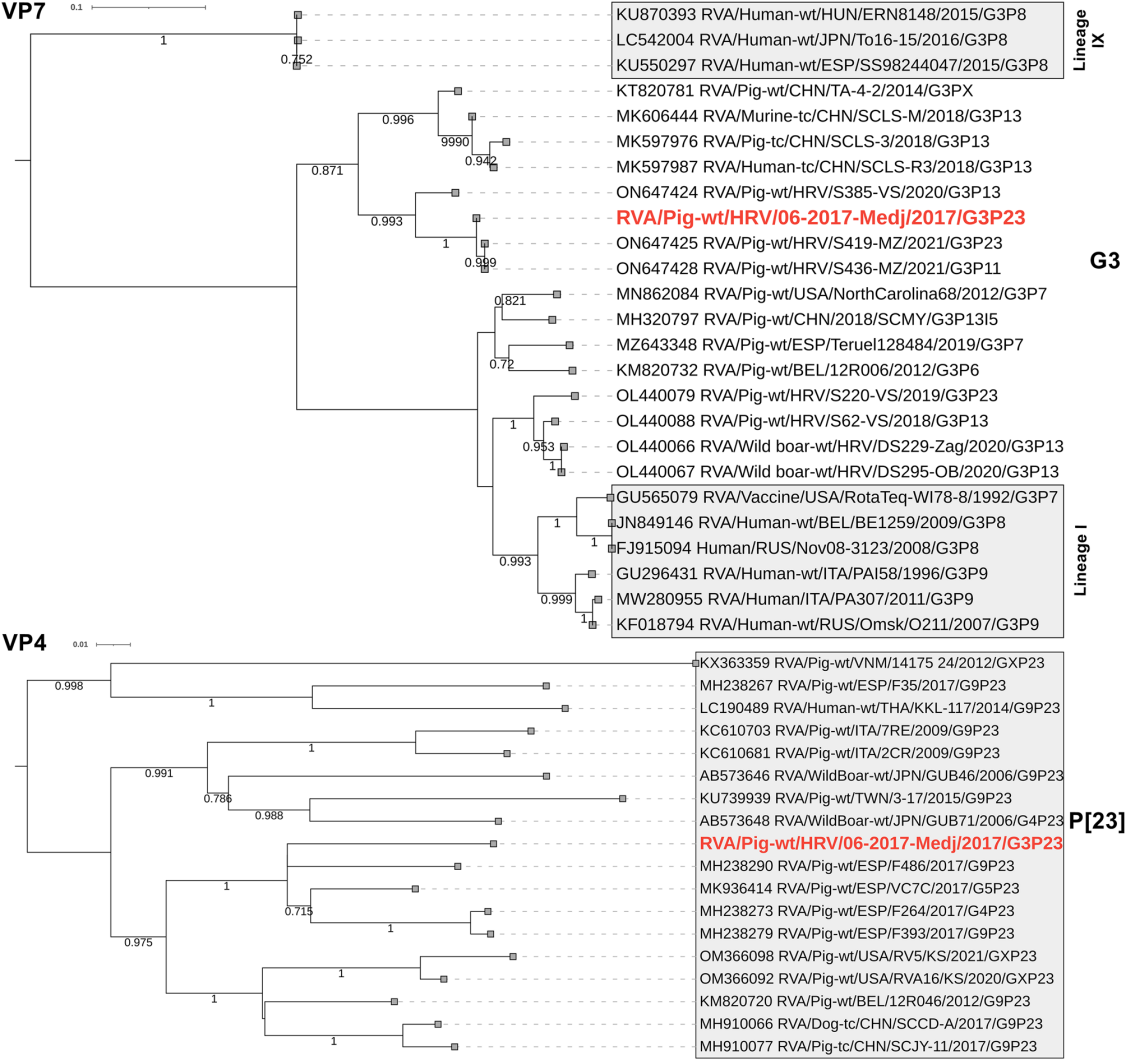
Phylogenetic relationship between RVA strains based on the VP7 (**A**) and VP4 (**B**) genomic segments. The strain described in the present study is bolded and marked in red. All sequences are representative of the genotypes G3 (**A**) and P[23] (**B**). Human G3 lineages (Lineages I and IX) are annotated. The GenBank accession numbers of referent strains are designated within taxa. Based on the complete CDS, both trees were generated by the ML method and T92 + G + I model in MEGA 11 software. The branching stability of each phylogenetic tree was assessed by 1000 bootstrap replicates (values indicated adjacent to the nodes if > 0.7). The scale bar represents the number of substitutions per site. In displaying RVA strain nomenclature within taxa, the brackets for the P genotype were omitted for the sake of simplicity.

The RVA/Pig-wt/HRV/06-2017-Medj/2017/G3P[23] strain was likely responsible for the second outbreak in June 2017 (most mapped reads on NGS and positive IHC staining). The complete genotype constellation G3-P[23]-I5-R1-C1-M1-A8-N1-T1-E1-H1 was revealed. Furthermore, the phylogenetic analysis confirmed this strain to be a typical porcine RVA in all genomic segments (Fig. 3, Supplementary Fig. S1). Sequence identities to the closest phylogenetic clade representatives are shown in Table 1.

**Table 1:** *Rotavirus A* and *Rotavirus B* nucleotide and amino acid sequence identities to the phylogenetically closest referent strains.

Genomic segment	*Rotavirus A*				*Rotavirus B*			
	% Cutoff^a^	Genotype	% nt identity	% aa identity	% Cutoff^a^	Genotype	% nt identity	% aa identity
VP7	80	G3	87.1–88.9	94.4–96.1	80	**G27**	74.1–76.7	76.6–84.2
VP4	80	P[23]	85.2–92.8	92.7–96	80	**P[6]**	76.5–76.7	82.7–82.8
VP6	85	I5	91.6–94.8	95.7–97.9	81	I9	82.2–85.8	91–93.3
VP1	83	R1	85.6–93.4	96.4–98.1	78	**R7**	79.2–81.4^b^	86.7–90.8
VP2	84	C1	88.3–94.6	97.3–98.9	79	**C6**	79–80.2^c^	89.9–90.9
VP3	81	M1	89–93.4	93.6–97.7	77	M4	78.4–84	86.1–90.1
NSP1	79	A8	85.9–92.4	88–94.2	76	A8	81.1–85.3	75.2–92.5
NSP2	85	N1	88.7–96	95.5–98.4	83	N10	83.9–86.8	93.3–97
NSP3	85	T1	87.4–91.5	91.6–97.7	78	T4	78.5–82.6	80–83.6
NSP4	85	E1	88.4–96.2	92–99.4	76	E4	77.6–85.1	75.6–84.6
NSP5	91	H1	92.9–98.6	94.4–99.4	79	H7	77.3–83^d^	76.4–86.2

RVB genotypes in boldface mark a proposed novel genotype.

^a^Previously defined cutoff value for all 11 RVA^18^ and RVB^36^ genomic segments.

^b^The nucleotide identity % values to the representatives of the newly proposed R7 genotype. The cutoff value of 79% was set in contrast to the previously defined 78%.

^c^The nucleotide identity % values to the representatives of the newly proposed C6 genotype.

^d^RVB strains (MG275052, MK953197 and KR052713), sharing lower than cutoff identity values, cluster with other H7 representatives, lacking evident pattern (Supplementary Fig. S2).

**Figure 4:**
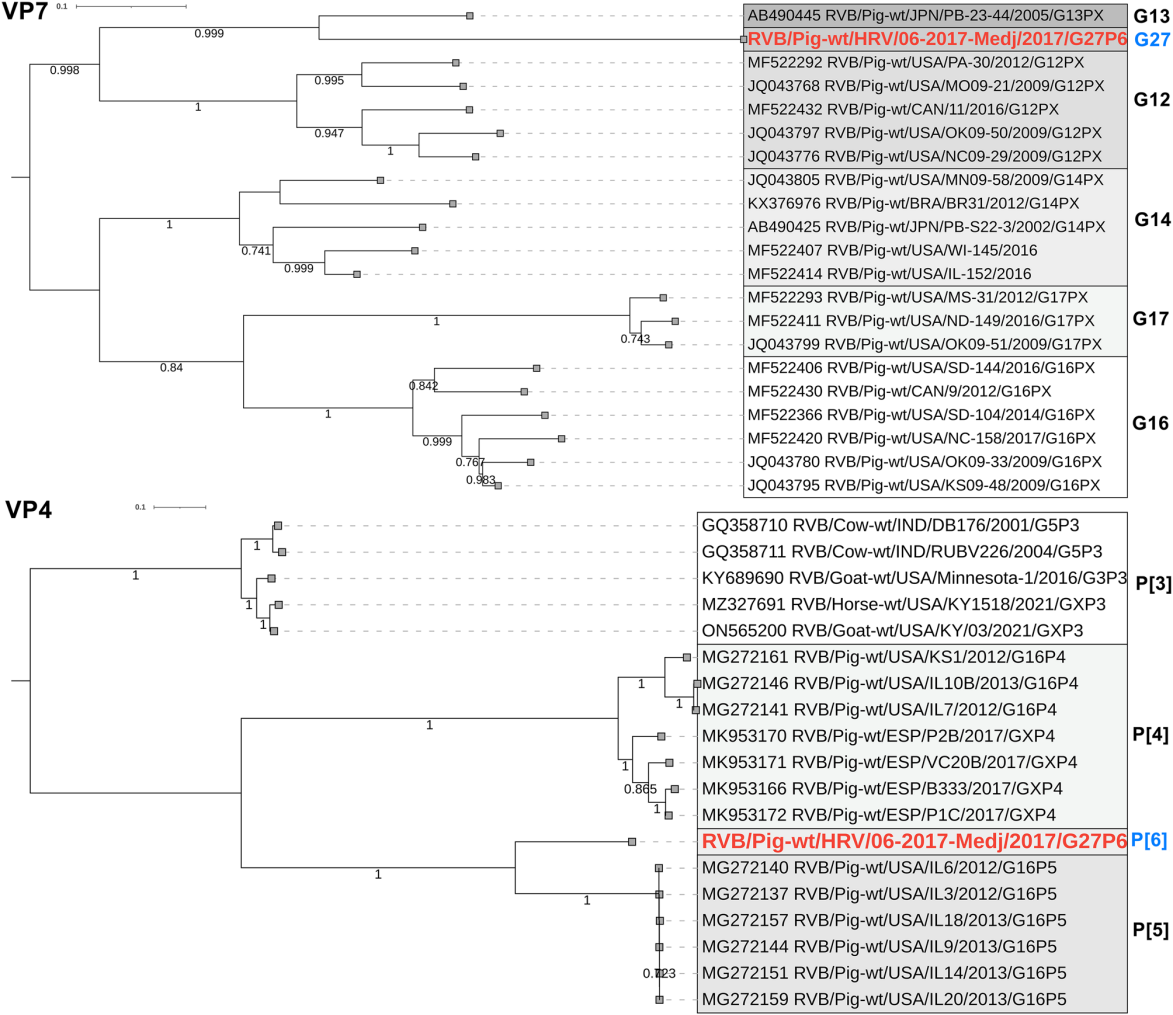
Phylogenetic relationship between RVB strains based on the VP7 (**A**) and VP4 (**B**) genomic segments. The strain described in the present study is bolded and marked in red, and corresponding novel genotypes G27 and P[6] are annotated in blue. The GenBank accession numbers of referent strains are designated within taxa. Based on the complete CDS, both trees were generated by the ML method and T92 + G + I (VP7) or HKY + G + I (VP4) model in MEGA 11 software. The branching stability of each phylogenetic tree was assessed by 1000 bootstrap replicates (values indicated adjacent to the nodes if > 0.7). The scale bar represents the number of substitutions per site. In displaying RVB strain nomenclature within taxa, the brackets for the P genotype were omitted for the sake of simplicity.

Compared to the RVA, the lower read numbers were mapped to reference RVB and PKV genomes. For the RVB/Pig-wt/HRV/06-2017-Medj/2017/G27P[6] strain, the complete genotype constellation G27-P[6]-I9-R7-C6-M4-A8-N10-T4-E4-H7 was assembled. Sequence identities to the closest phylogenetic clade representatives are shown in Table 1. The results of the phylogenetic analysis confirm the porcine origin of all genomic segments (Fig. 4, Supplementary Fig. S2). As an important finding, we are proposing the novel genotypes for the VP7 (G27), VP4 (P[6]), VP1 (R7) and VP2 (C6) genomic segments (Fig. 4, Supplementary Fig. S2). While a designation of proposed novel genotypes for VP7, VP4 and VP2 aligns with the current genotype classification^36^, we suggest modification for the VP1 segment. The current RVB strain shared 76.5–79% nt identity with many R4 strains, while the VP1 cutoff is 78%^36^. By increasing the cutoff to 79%, we propose dividing the R4 genotype into three different genotypes (R4 and novel R6 and R7) with excellent bootstrap support (Supplementary Fig. S2). In addition to the evolutionary distinctiveness of the Croatian G27P[6] RVB strain, its heterogeneity was further driven by insertions observed in the NSP4 segment; more precisely, three amino acids (KDK) were inserted at position 194 of the aa sequence.

**Figure 5:**
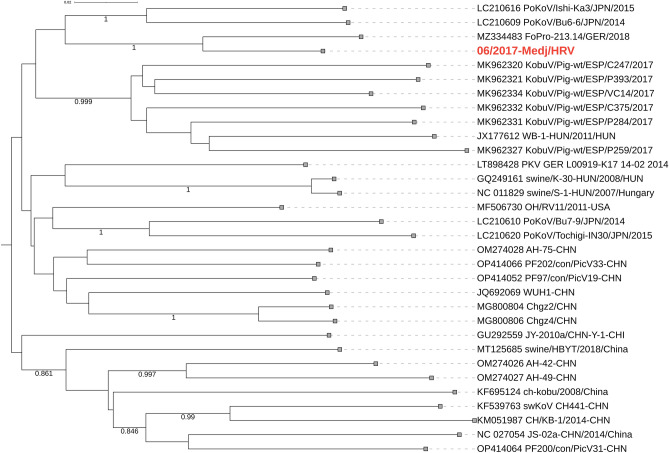
Phylogenetic relationship between PKV strains based on the complete genome nucleotide sequence. The strain described in the present study is bolded and marked in red. The GenBank accession numbers of referent strains are designated within taxa. The tree was generated by the ML method and GTR + G + I model in MEGA 11 software. The branching stability of each phylogenetic tree was assessed by 1000 bootstrap replicates (values indicated adjacent to the nodes if > 0.7). The scale bar represents the number of substitutions per site.

The complete genome of PKV was also revealed with the phylogenetically closest German PKV strain from 2018 (MZ334483) (Fig. 5), sharing 93.2% and 98.5% nt and aa identity, respectively. Nucleotide identities to all other strains were below 90% (87.2–89.9). Distinctive clustering was not evident (low bootstrap values) (Fig. 5).

### Follow-up study

The results revealed the circulation of PEDV from January to March 2017. All other samples from June 2017 until January 2021 tested PEDV and TGEV negative. RVA was detected throughout the study, proving its endemic nature. Overall it was detected in 58.2% of samples (46/79), 52.2% (35/67) in diarrhoeic, and 91.7% (11/12) in healthy pigs. Six different G (G1, G2, G3, G5, G6 and G9) and P (P[7], P[8], P[11], P[13], P[23] and P[32]) RVA genotypes in 13 genotype combinations were found circulating, respectively^20^. G3P[23] was present in all sampling seasons^20^. Although the NGS detected RVB in June 2017, it has been present since January 2017, only in diarrhoeic pigs. Eight out of 12 samples directly related to outbreaks in 2017 were RVB-positive. It was further detected in 2018 (5/10) and 2019 (1/27) with decreasing prevalence. The last RVB-positive sample was detected in July 2019, and the overall prevalence was 17.7% (14/79). PKV was established as the most prevalent (84.8%, 67/79) viral pathogen. Considering diarrhoeic vs non-diarrhoeic pigs, it was detected in 85.1% (57/67) and 83.3% (10/12), respectively. The specific binding of the RVB and PKV primer sets was confirmed by Sanger sequencing. Successful detection of IPC RNA excluded the presence of PCR inhibitors in all tested samples.

### The impact of diarrhoea outbreaks on farm production results

**Figure 6:**
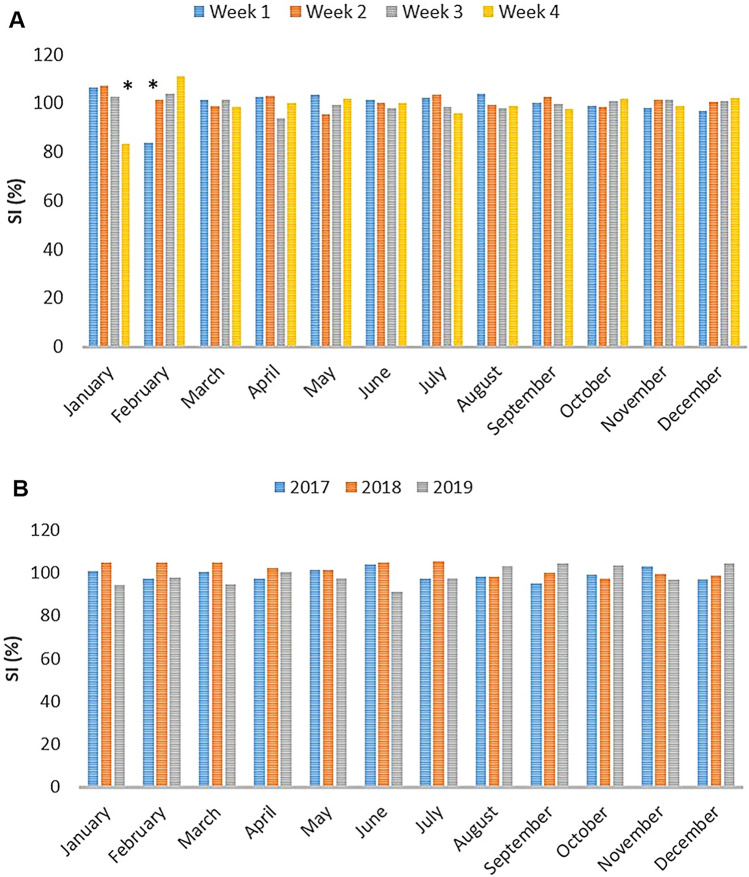
Seasonal indices (SI) for the average number of weaned pigs per saw in 2017–2019 timeframe. The SI (%) was calculated by comparing the average number of weaned pigs for every saw in each week (**A**) or month (**B**) to the average number in the respective week for all months (**A**) or in the respective month for all three years (**B**). A significant decrease in the number of weaned pigs per sow is indicated with an asterisk. All months were divided into four weeks, and four additional weeks over the year were added to the nearest week, and the average was calculated.

The results of SI calculation clearly show that the 1st outbreak involving PEDV had only a minor impact on the number of weaned pigs per sow, which was observed in the 4th week of January (− 15.46%) and in the 1st week of February 2017 (− 20.64%) (Fig. 6A). In contrast, outbreak 2, reported in June 2017, had no significant effect on the individual production results (Fig. 6A). On a monthly basis, in 2017, no negative impact was observed (Fig. 6B) for both outbreaks. In subsequent years, no significant changes in the number of weaned pigs per sow were observed (Fig. 6B). Seasonal index calculations showed irregular fluctuations in piglet mortalities and newborns per sow (data not shown). The farrowing index slightly decreased with increasing number of sows: 2.30, 2.27 and 2.20 per sow in 2017, 2018 and 2019, respectively.

Seasonal influence, i.e. irregular periodic fluctuations, was not statistically significant for the number of weaned pigs per sow (*p* < 0.389). Still, it had an impact on mortalities (*p* < 0.026) and newborns (*p* < 0.006). More importantly, the forecasted trend was significantly positive in terms of the number of weaned pigs (*p* < 0.024) and newborns per sow (*p* < 0.0001) and favourably negative with respect to piglet mortality per sow (*p* < 0.0001) (Supplementary Fig. S3). The fit of the ARIMA method was very good, with the residuals showing approximately equal variability along the length of the series and showing no sign of trend or shift.

## Discussion

The present study brings new data on the complexity of underlying viral pathogens involved in two diarrhoea outbreaks and their impact on selected production results in a large farrow-to-finish holding. The first outbreak in January 2017 was caused by the PEDV-SeCoV S gene recombinant strain (Fig. 2). Similar PEDV-SeCoV recombinants were involved in diarrhoea outbreaks in Hungary^47^, Slovenia (GenBank), Poland^14^, France (GenBank), The Netherlands (GenBank), Spain^11,13^ and Germany^48^ (Fig. 2). In addition to conventional molecular methods and NGS, a direct connection to a clinical disease was demonstrated with evident IHC staining of PEDV-SeCoV in enterocytes of affected villi in the small intestine (Fig. 1). Trade of live pigs has been associated with the global transmission of PEDV^49^, which was a probable cause for PEDV-SeCoV introduction into the study holding. The first case of PEDV in Croatia was confirmed a year earlier, in 2016, but that was a typical S-INDEL strain^6^ (Fig. 2). Subsequent serological testing in early 2017, revealed the circulation of PEDV in one of the largest pig producers in Croatia which reported significant losses^6,50^. Unfortunately, the sequence of the responsible PEDV strain remained unknown^6^. Although the outbreak reported here coincides in time with that outbreak, it is tempting to speculate that different PEDV variants caused these outbreaks due to significant impact variations. The PEDV-SeCoV recombinant strain described in the present study caused a relatively mild impact outbreak. To the best of our knowledge, our study brings a comprehensive statistical background to this claim for the first time, revealing only a short-lasting disturbance in the number of weaned pigs per sow on a weekly basis (Fig. 6). The only control measure the holding implemented was feedback to sows and gilts in gestation. The stabilisation and improvement were visible approximately ten days later.

The second outbreak in June 2017 was even less impactful since no statistically significant disturbance in selected production metrics was observed (Fig. 6). Our investigation revealed that viral agents were also behind the aetiology of that outbreak, but this time, mixed infection with RVA, RVB and PKV. Since RVA was the most abundant considering the NGS results and was further confirmed by IHC in enterocytes of the small intestine’s villi (not shown) and crypts of Lieberkűhn in the colon (Fig. 1), we believe it was the principal causative agent of diarrhoea. However, RVB and PKV might have synergistically contributed to the clinical disease of affected piglets. Since our study did not aim to discover all possible diarrhoea-causing viral pathogens in addition to those revealed by NGS in selected samples, the impact of some other porcine enteric viruses, such as *Rotavirus C*, astroviruses and sapoviruses, might have been underestimated.

The genotype constellation of the RVA strain responsible for the diarrhoea outbreak was a typical porcine G3-P[23]-I5-R1-C1-M1-A8-N1-T1-E1-H1, confirmed by phylogenetic analysis of all genomic segments (Fig. 3, Supplementary Fig. S1). In contrast to the outbreak connection, the follow-up study showed that RVA was less prevalent in diarrhoeic than in healthy pigs. Nevertheless, a small sample size of healthy pigs precludes relevant conclusions. Moreover, our comprehensive analysis of RVA in domestic pigs, which involved the samples from the current follow-up study, confirmed a statistically significant connection between RVA and diarrhoea^20^. Like many others^15^, this study demonstrates the endemic nature of RVA infections in pig holdings.

Even though we have detected RVB in a mixed infection with RVA and PKV, it was recently described as solely capable of inducing neonatal diarrhoea outbreaks in domestic pigs^51^. Since RVB was detected in lower abundance on NGS than RVA, and IHC staining was unsuccessful despite optimisation efforts, we could not define its involvement in the second diarrhoea outbreak. Still, the frequent detection of RVB during outbreaks in 2017, with decreasing prevalence until July 2019 (detected only in diarrhoeic pigs), advocates its potential involvement in the aetiology of diarrhoea. However, a primer pair designed to amplify the specific RVB strain may have impaired its diagnostic sensitivity to other non-I9-genotype strains. Our study on the whole genome characterisation of RVB brings new knowledge on its genetic diversity in domestic pigs. The underlying genotype constellation was the porcine-related G27-P[6]-I9-R7-C6-M4-A8-N10-T4-E4-H7. Four novel genotypes were suggested (G27, P[6], R7 and C6), and many others were phylogenetically distant (Fig. 4, Supplementary Fig. S2), which is in line with the recent claim that RVB evolves more rapidly in domestic pigs compared to other hosts^36^. However, a general understudy and the lack of complete or even partial RVB genomes in available genetic depositories make such claims potentially premature. Additionally, our study accentuates a developmental phase of RVB classification, suggesting modification in the recently established VP1 nucleotide cutoff^36^. RVA^18^ and RVC^52^ nucleotide cutoff values are generally higher for all segments except for the RVA’s VP7 and the VP4.

Porcine kobuvirus was the least abundant on NGS but highly prevalent in the follow-up study, both in diarrhoeic and healthy pigs, proving its endemicity. These results support the statement that its involvement in diarrhoea aetiology in pigs is inconclusive^25,53,54^. However, pathogenic synergy in mixed infections cannot be excluded. The phylogenetically closest was the German strain^55^, but there is a general shortage of whole PKV genomes originating in Europe for better phylogeographical comparison (Fig. 5).

Although both diarrhoea outbreaks did not cause significant disturbance in the studied farrow-to-finish holding, and statistical forecasting confirmed positive trends regarding mortality, newborns and weaned pigs per sow, many additional production results (e.g. growth retardation, extended fattening and treatment costs) might have been impacted^9^. Unfortunately, these data were not available for statistical analysis. The farrowing index decreased in three consecutive years whilst the number of sows increased. Possible reasons are diverse, but the restricted capacity of gestation pens largely contributed to reduced welfare, potentially impacting the conception rate. Nevertheless, that was compensated with larger litter size and decreasing mortalities, contributing to positive trends and forecasts.

To conclude, this study provided an insightful view into the background complexity of diarrhoea-causing viral agents in large farrow-to-finish holding and their impact on specific production metrics. Whole genome characterisation of PEDV-SeCoV recombinant, RVA, RVB and PKV strains generated an in-depth look into genome specificities and phylogenetic relationships. The endemicity was corroborated for RVA and PKV, in contrast to the PEDV-SeCoV recombinant strain. Punctual, comprehensive and timely investigation of diarrhoea outbreaks is a prerequisite for applying adequate pig health and biosecurity management. Calculating the impact such outbreaks had on specific production metrics in the past can potentially shape decisions on management improvements in the future.

### Supplementary Information

Supplementary Figure S1.

Supplementary Figure S2.

Supplementary Figure S3.

Supplementary Legends.

## Data Availability

Sequences of virus strains characterised in the present study are deposited to the GenBank under accession numbers: OQ302146 (PEDV), OQ333046-OQ333056 (RVA), OQ506616-OQ506626 (RVB) and OQ595081 (PKV).
