# Supplementary figures and images for: The impact and complete genome characterisation of viruses involved in outbreaks of gastroenteritis in a farrow-to-finish holding
Source: Sci Rep. 2023 Oct 31;13:18780. doi: 10.1038/s41598-023-45994-4 (PMC10618538; doi:10.1038/s41598-023-45994-4)

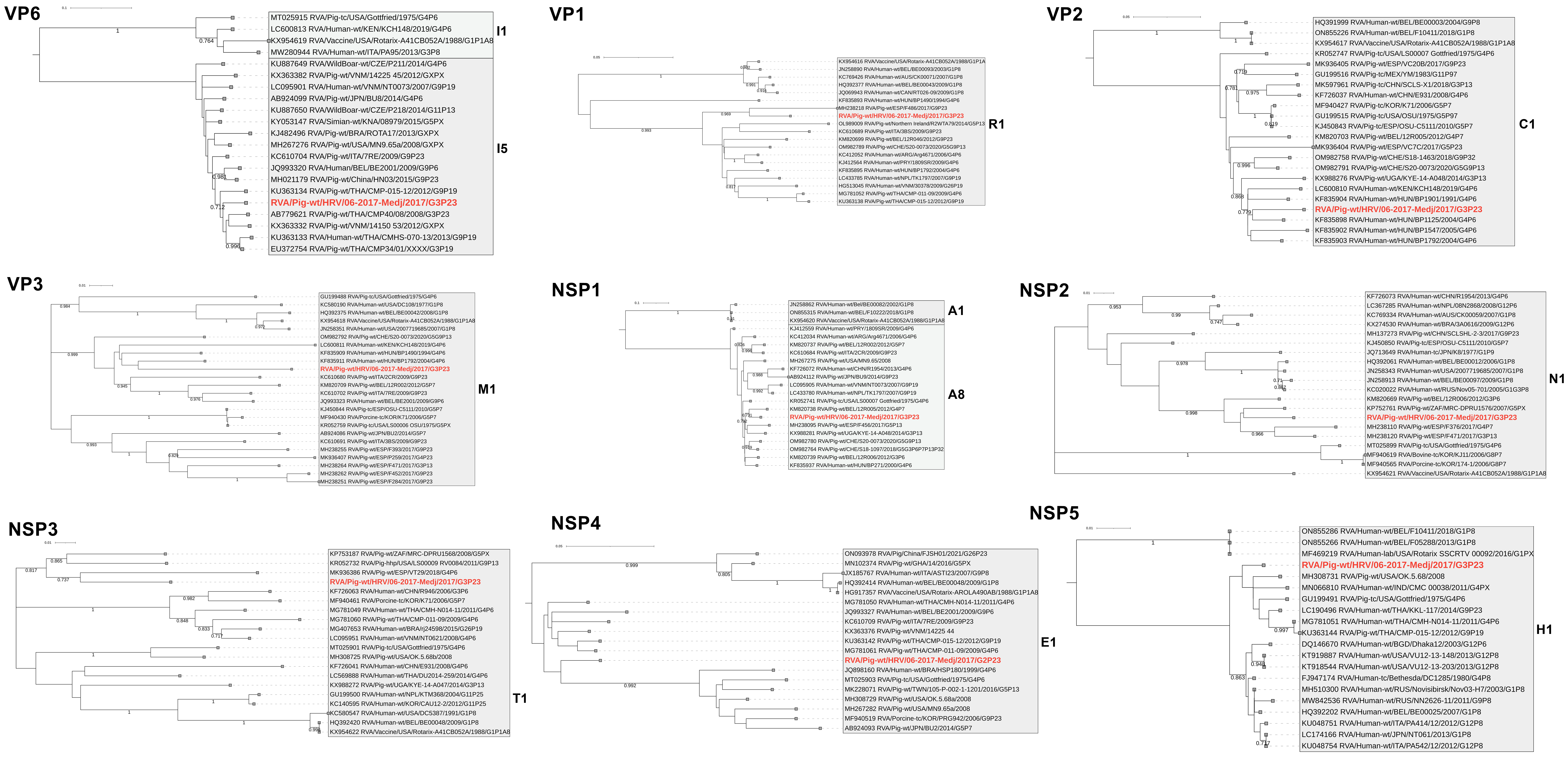

Supplement: Supplementary file 1 — Supplementary Figure S1. [file 41598_2023_45994_MOESM1_ESM.tif]

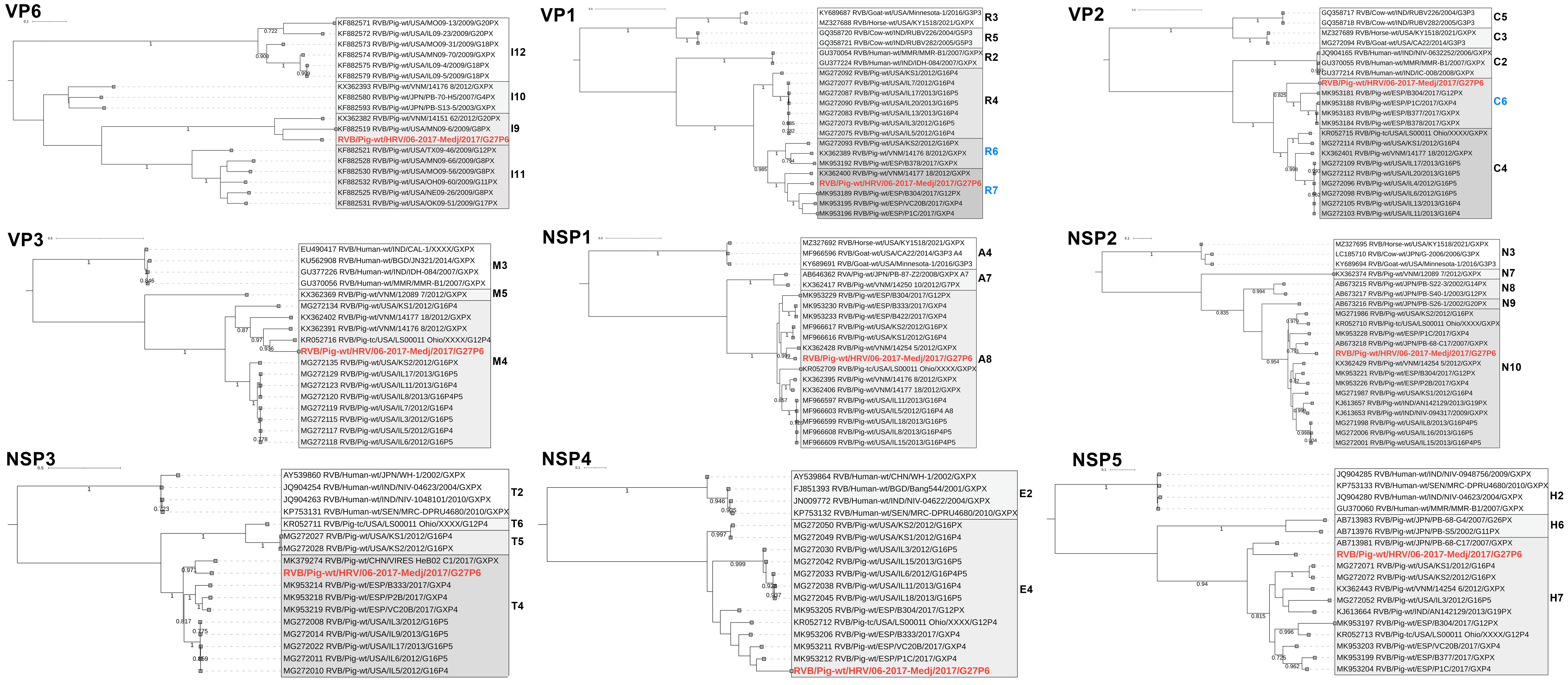

Supplement: Supplementary file 2 — Supplementary Figure S2. [file 41598_2023_45994_MOESM2_ESM.tif]

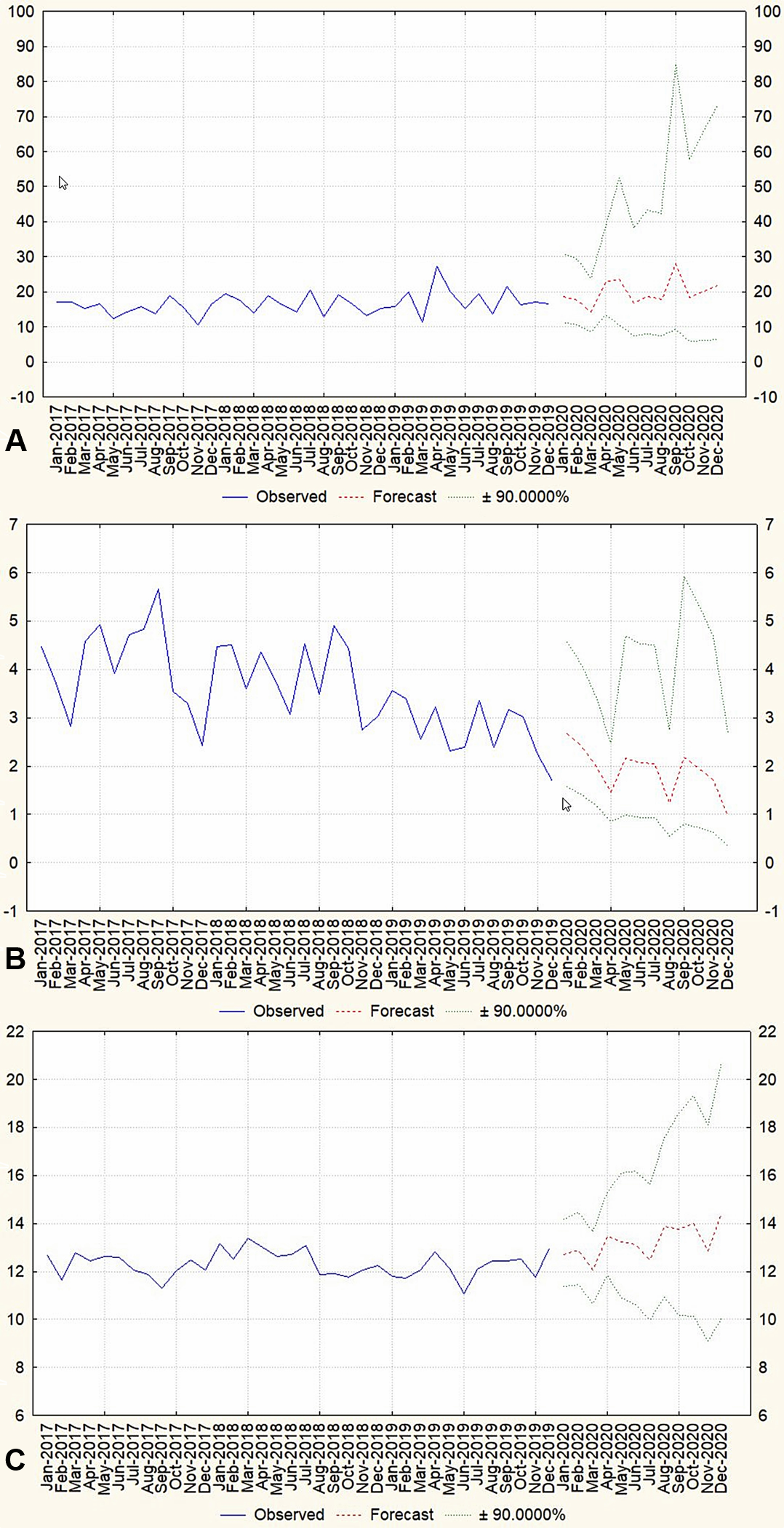

Supplement: Supplementary file 3 — Supplementary Figure S3. [file 41598_2023_45994_MOESM3_ESM.tif]
