# Supplementary material for: The impact and complete genome characterisation of viruses involved in outbreaks of gastroenteritis in a farrow-to-finish holding
Source: Sci Rep. 2023 Oct 31;13:18780. doi: 10.1038/s41598-023-45994-4 (PMC10618538; doi:10.1038/s41598-023-45994-4)
Supplement: Supplementary file 4 — Supplementary Legends. [file 41598_2023_45994_MOESM4_ESM.docx]

**Supplementary information**

**Supplementary Fig. S1. Phylogenetic relationship between RVA strains based on the VP6, VP1-VP3 and NSP1-NSP5 genomic segments.** The strain described in the present study is bolded and marked in red. Corresponding genotypes are annotated for each RVA segment. The GenBank accession numbers of referent strains are designated within taxa. Based on the complete CDS, trees were generated in MEGA 11 software by the ML method and T92+G (VP6, NSP2-NSP5), T92+G+I (NSP1), TN93+G+I (VP1), TN93+G (VP2) or GTR+G+I (VP3) model. The branching stability of each phylogenetic tree was assessed by 1000 bootstrap replicates (values indicated adjacent to the nodes if > 0.7). The scale bar represents the number of substitutions per site. In displaying RVA strain nomenclature within taxa, the brackets for the P genotype were omitted for the sake of simplicity.

**Supplementary Fig. S2. Phylogenetic relationship between RVB strains based on the VP6, VP1-VP3 and NSP1-NSP5 genomic segments**. The strain described in the present study is bolded and marked in red, and novel genotypes R6, R7 and C6 are annotated in blue. The GenBank accession numbers of referent strains are designated within taxa. Based on the complete CDS (VP2 was near complete), trees were generated in MEGA 11 software by the ML method and T92+G (NSP2), HKY+G (NSP4), TN93+G+I (VP6 and NSP3), TN93+G (NSP5) or GTR+G+I (VP1-VP3 and NSP1) model. The branching stability of each phylogenetic tree was assessed by 1000 bootstrap replicates (values indicated adjacent to the nodes if > 0.7). The scale bar represents the number of substitutions per site. In displaying RVB strain nomenclature within taxa, the brackets for the P genotype were omitted for the sake of simplicity.

**Supplementary Fig. S3.** **The trend and forecast analysis plot of selected production metrics in a large farrow-to-finish holding.** The plot shows a trend of the observed data of newborns (A), piglet mortality (B) and weaned pigs per sow (C) during 2017-2019 and the forecast for one year ahead (2020). The analysis was performed using the ARIMA method, which contains three parameters in the prediction equation: the number of autoregressive terms, nonseasonal differences, and lagged forecast errors.
